# Efficient soluble expression of disulfide bonded proteins in the cytoplasm of *Escherichia coli* in fed-batch fermentations on chemically defined minimal media

**DOI:** 10.1186/s12934-017-0721-x

**Published:** 2017-06-15

**Authors:** Anna Gąciarz, Narendar Kumar Khatri, M. Lourdes Velez-Suberbie, Mirva J. Saaranen, Yuko Uchida, Eli Keshavarz-Moore, Lloyd W. Ruddock

**Affiliations:** 1 Faculty of Biochemistry and Molecular Medicine, University of Oulu, P.O. Box 5400, 90014 Oulu, Finland; 2 The Department of Process and Environment Engineering, University of Oulu, P.O. Box 8000, 90014 Oulu, Finland; 3 The Advanced Center for Biochemical Engineering, Department of Biochemical Engineering, University College London, Bernard Katz Building, Gower Street, London, WC1E 6BT UK

**Keywords:** Disulfide bonds, Cytoplasm, *Escherichia coli*, Fermentation, Fed-batch, Interleukin 6, Growth hormone, scFv, Avidin

## Abstract

**Background:**

The production of recombinant proteins containing disulfide bonds in *Escherichia coli* is challenging. In most cases the protein of interest needs to be either targeted to the oxidizing periplasm or expressed in the cytoplasm in the form of inclusion bodies, then solubilized and re-folded in vitro. Both of these approaches have limitations. Previously we showed that soluble expression of disulfide bonded proteins in the cytoplasm of *E. coli* is possible at shake flask scale with a system, known as CyDisCo, which is based on co-expression of a protein of interest along with a sulfhydryl oxidase and a disulfide bond isomerase. With CyDisCo it is possible to produce disulfide bonded proteins in the presence of intact reducing pathways in the cytoplasm.

**Results:**

Here we scaled up production of four disulfide bonded proteins to stirred tank bioreactors and achieved high cell densities and protein yields in glucose fed-batch fermentations, using an *E. coli* strain (BW25113) with the cytoplasmic reducing pathways intact. Even without process optimization production of purified human single chain IgA_1_ antibody fragment reached 139 mg/L and hen avidin 71 mg/L, while purified yields of human growth hormone 1 and interleukin 6 were around 1 g/L. Preliminary results show that human growth hormone 1 was also efficiently produced in fermentations of W3110 strain and when glucose was replaced with glycerol as the carbon source.

**Conclusions:**

Our results show for the first time that efficient production of high yields of soluble disulfide bonded proteins in the cytoplasm of *E. coli* with the reducing pathways intact is feasible to scale-up to bioreactor cultivations on chemically defined minimal media.

**Electronic supplementary material:**

The online version of this article (doi:10.1186/s12934-017-0721-x) contains supplementary material, which is available to authorized users.

## Background

The production of recombinant proteins in* Escherichia coli* has a number of advantages over other systems including fast growth, well characterized genetics, high productivity and an organism that is Generally Recognized As Safe (GRAS). However, expression of homogenously folded proteins containing post-translational modifications such as disulfide bonds is challenging. Disulfide bonds are covalent linkages that are essential for the native structure and biological activity of many secreted and outer membrane proteins [1]. In natural systems disulfide bonds are synthesized in cellular compartments that are able to maintain an oxidizing environment e.g. the endoplasmic reticulum in eukaryotes and the periplasm of Gram-negative bacteria. In most cellular compartments, disulfide bonds are synthesized de novo by a sulfhydryl oxidase that oxidizes cysteine thiols; additionally, proteins with multiple disulfide bonds need a disulfide bond isomerase that rearranges randomly oxidized disulfides to their native configuration [2]. In contrast, the cytoplasm contains multiple pathways for the reduction of disulfide bonds and these are crucial for catalytic turnover of cytoplasmic enzymes such as ribonucleotide reductase [3].

The most common way to deal with such difficult-to-express proteins in *E. coli* on an industrial scale is either to target the protein to the periplasm or to synthesize the protein of interest (POI) as inclusion bodies in the cytoplasm and subsequently solubilize and refold in vitro [4].

*Escherichia coli* hosts enzymes that catalyze disulfide bond formation in the periplasm, therefore a recombinant protein can be targeted to and potentially fold in this compartment. However, the periplasm constitutes only 8 to 16% of the total bacterial cell volume [5]; moreover, heterologous proteins need a signal sequence on the N-terminus to be exported to the periplasm and there is only a limited number of transporters that allow proteins to cross the cytoplasmic membrane and they can easily become overloaded [6]. These two factors combine to result in typically low protein yields upon periplasmic expression unless extensive optimization of production processes is undertaken.

In contrast, the cytoplasm of *E. coli* has a high capacity for accumulating recombinant proteins, which can exceed 30% of the total cellular protein [4]. Therefore, many marketed pharmaceuticals produced in *E. coli* are produced as inclusion bodies [7, 8]. Inclusion bodies are formed when a protein emerging from the ribosome is unfolded or misfolded and hence are likely to aggregate. Although inclusion bodies can be produced in high yields, are easily physically separated from other cellular components and are resistant to cellular proteases [8], their use is problematic as elaborate in vitro solubilization, refolding and purification procedures are required to recover biologically active protein. Furthermore, refolding conditions need to be optimized for each POI and in many cases only 15–25% of inclusion bodies will be converted to bioactive product [9]. In addition, separation of correctly folded from partially folded POI can be very problematic as they can have very similar biophysical properties.

Although several strategies have been developed to promote biosynthesis of soluble proteins in the cytoplasm of *E. coli*, including expression at lower temperatures (15–23 °C), co-expression of molecular chaperones, protein engineering and attachment of fusion tags, modification of growth media [8], they are very often inefficient for production of proteins with disulfide bonds and/or are infeasible for larger scale production [10]. Engineering attempts to create strains specifically dedicated for cytoplasmic expression of proteins containing disulfide bonds have been based on the disruption of genes of reducing cytoplasmic enzymes [11]. In commercially available strains, such as Origami (Novagen) and SHuffle (New England Biolabs), the thioredoxin reductase (*trxB*) and glutathione reductase (*gor*) genes are deleted. The SHuffle strain additionally expresses the bacterial disulfide bond isomerase DsbC in the cytoplasm. However the viability of these strains relies on another mutation located in peroxiredoxin *aphC* gene that suppresses redox deficiencies [12]. The yield of recombinant disulfide-bonded proteins obtained in redox engineered strains is usually low and still often requires assistance of molecular chaperones [13] or extensive optimization of growth conditions e.g. temperature, rich media, time and strength of induction, etc. [14]. Although engineered strains are well marketed, there is no evidence of their efficient soluble protein productivity at bioreactor scale in chemically defined minimal media since they require the addition of yeast extract and soytone [15].

The issues in both periplasmic and cytoplasmic expression of disulfide bond containing proteins suggests a need for an alternative system for large scale production in *E. coli*, in particular one which will allow production in chemically defined minimal media.

Based on the mechanisms for natural disulfide bond formation in other cellular compartments we have developed a system called CyDisCo (cytoplasmic disulfide bond formation in *E. coli*), in which soluble expression of a recombinant protein containing disulfide bonds in the cytoplasm of *E. coli* is possible thanks to pre-expression or co-expression of a sulfhydryl oxidase (usually Erv1p from *Saccharomyces cerevisiae*) and a disulfide bond isomerase (usually human PDI [16, 17]). While Δ*trxB*/Δ*gor* strains cannot be grown in chemically defined minimal media, probably due to the role of the reducing pathways in processes such as DNA synthesis through catalytic turnover of ribonucleotide reductase [3], the CyDisCo system does not require disruption of the reducing pathways and hence it should in theory be possible to use it in chemically defined minimal media. In addition the CyDisCo system is easily transferred between expression hosts [16–19].

In this work we undertook proof of concept studies to see if it is possible to produce disulfide bond containing proteins in chemically defined minimal media using the CyDisCo system. We tested expression in fed-batch fermentation of three human proteins, whose native structure is stabilized by disulfide bonds: growth hormone 1 (GH1), interleukin 6 (IL-6) and a single chain variable fragment (scFv), as well as chicken avidin.

## Results

CyDisCo has previously been shown to allow efficient cytoplasmic production of a range of disulfide bond containing proteins, including GH1, interleukins and antibody fragments such as scFv, in both deep well plates and shake flasks [16–19]. Unlike Δ*trxB/*Δ*gor* strains such as Origami CyDisCo has no requirement to disrupt the reducing pathways in the cytoplasm and this should allow growth in defined minimal media. We therefore hypothesized that CyDisCo should be amenable to large scale cultivation e.g. in fed-batch fermentation. Here we tested that hypothesis with fermentation scale growth of two proteins which have previously been shown to work in shake flask scale (human GH1 [19] and an IgA_1_ based scFv [17]) as well as human IL-6 and chicken avidin.

CyDisCo has a number of formats. Those that involve the use of transmembrane catalysts i.e. inverted VKOR or inverted DsbB [20] require more optimization as too high level of over-expression of the transmembrane component is deleterious to the host while too little results in inefficient disulfide bond formation. In contrast the CyDisCo variants based on Erv1p are well tolerated.

Here CyDisCo has been applied in either a one- or two-plasmid based format. GH1, IL-6 and avidin were expressed from a one-plasmid based format, specifically a polycistronic modified pET23-based vector with a Ptac promoter that carries also genes for parallel synthesis of Erv1p (*Saccharomyces cerevisiae*) and PDI (human). scFv IgA_1_ was expressed using a two-plasmid format with the scFv expressed from a pET23-based plasmid with a Ptac promoter, while CyDisCo components i.e. Erv1p and PDI are co-expressed from the second, pLysS-based vector, under a Ptac promoter and with a p15A origin of replication. Both CyDisCo formats are functional in fed-batch fermentation on minimal defined media (see below).

Initial proof of concept cultivations were performed in 1 L stirred tank reactors in fed-batch cultures grown on minimal defined media with glucose as the carbon source. Protein production was induced during exponential growth phase. The cultures achieved good cell densities, with cell dry weight (CDW) up to 28.5 g/L, indicating that cell growth is not impaired by the expression of CyDisCo components. Higher cell densities could potentially be reached in an optimized process.

**Fig. 1:**
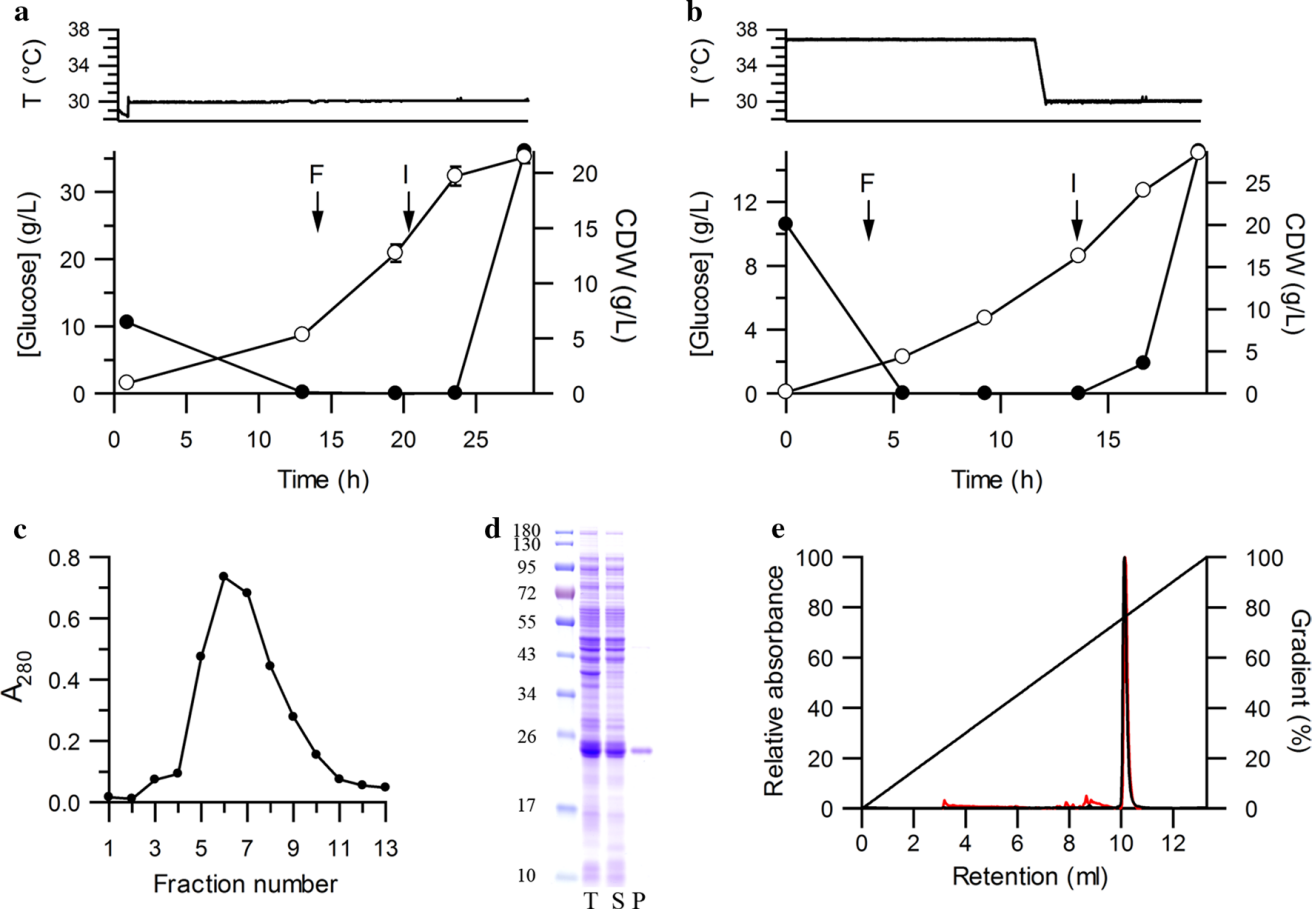
Production of GH1. **a**, **b** Representative growth profiles of K-12 strain BW25113 co-expressing coPDI, coErv1p and GH1; **a** constant temperature growth, **b** growth at 37 °C to increase cell mass, then shift to 30 °C pre-induction. *Error bars* represent the standard deviation from 3 samples. *F* indicates feeding started, *I* indicates induction. Cells were harvested and protein purified from the last time point shown. **c** Representative elution profile from IMAC purification of GH1. **d** Coomassie stained reducing SDS-PAGE analysis of produced proteins: molecular marker, total *E. coli* lysate (T), soluble protein fraction (S) and IMAC purified GH1. **e** rpHPLC analysis of purified GH1: comparison of GH1 produced in fed-batch fermentation (*red*) and in shake flask (*black*)

Initial experiments focused on GH1 production in fed-batch fermentation in *E. coli* strain BW25113 (parental strain of the KEIO collection). Since Erv1p uses molecular oxygen, dissolved oxygen level was maintained at 30% of air saturation as is standard for *E. coli* fermentations rather than the low dissolved oxygen levels required for Δ*trxB/*Δ*gor* strains [15]. Since Erv1p does not tolerate 37 °C well, cultures were either grown at 30 °C (Fig. 1a) or grown initially at 37 °C to accumulate biomass, then the temperature was decreased to 30 °C for protein induction (Fig. 1b). Both methods produced soluble GH1 in high yields. The GH1 produced was purified by Immobilized Metal Affinity Chromatography (IMAC) followed by buffer exchange using a PD-10 gel filtration column to remove low molecular weight species which might absorb at 280 nm (Fig. 1c, d). The variability in purified protein yield between purifications from a single fermentation (9%) was comparable with the variability between fermentations (12%) indicating that the use of CyDisCo on fermentation scale is reproducible and robust. The yield of purified GH1 obtained was 0.97 ± 0.12 g/L (average 39 mg/g CDW).

GH1 purified from fermentation showed the expected molecular weight (Table 1) for the protein with disulfides present after treatment with N-ethylmaleimide, while an Ellman’s assay indicated 0.09 free thiol groups per protein. rpHPLC analysis of GH1 produced in fermentation showed a single species for GH1 and an identical profile to that produced in shake flask cultures (Fig. 1e). The presence of the correct number of disulfides, a single species by rpHPLC and high yields (implying protease resistance) indicates that CyDisCo can efficiently produce GH1 with native disulfide bonds in the cytoplasm of *E.coli* in fed-batch fermentation. To our knowledge this is the first evidence that disulfide bond containing proteins can be produced efficiently in the cytoplasm of *E. coli* in chemically defined minimal media.

**Table 1:** Analysis of free thiols content in purified proteins based on mass spectrometry and Ellman’s assay

Protein	Number of Cys	Expected mw (Da)	Experimental mw (Da)	Free thiols per molecule
GH1	4	23,210	23,211	0.09
IL-6	4	21,894	21,894	−0.01
ScFv IgA_1_	4	27,342	27,342	0.03
Avidin	2	15,426	15,426	N/D

The molecular weight of the purified proteins was determined after treatment with N-ethylmaleimide, which reacts with free thiol groups and adds 125 Da. Expected molecular weight assumes all cysteines are present as disulfides. No N-ethylmaleimide adducts were observed implying all cysteines were in disulfide bonds. The free thiol content of the purified proteins was determined by Ellman’s assay under denaturing conditions

*N/D* not determined

The use of Δ*trxB/*Δ*gor* strains not only requires the use of complex additives e.g. yeast extract, they also put limitations on the strain used. In contrast, CyDisCo can be freely transferred between strains and has worked in all B- and K-strains tested to date. Preliminary data indicates that this extends to fermentation scale growth (Additional file 1: Figure S1; strain W3110, yield of purified GH1 0.28 g/L; 18 mg/g CDW). In addition, preliminary data indicates that glycerol can be used in place of glucose as the carbon source with no loss of efficient production (Additional file 1: Figure S1; strain BW25113 yield of purified GH1 0.75 g/L; 37 mg/g CDW c.f. an average of 39 mg/g CDW with growth on glucose).

To ensure that the results obtained were not protein specific i.e. that CyDisCo has wider application in fed batch fermentation, the expression of other proteins was tested. Preliminary results with other proteins produced as per GH1, indicates that similar high yields can be obtained for IL-6 (Additional file 1: Figure S2, yield of purified IL-6 1.1 g/L; 41 mg/g CDW) while lower yields were obtained for chicken avidin (Additional file 1: Figure S3, yield of purified avidin 71 mg/L; 3.6 mg/g CDW).

The starting point for fermentation of a protein of interest i.e. prior to protein specific optimization varies considerably between research groups.

**Fig. 2:**
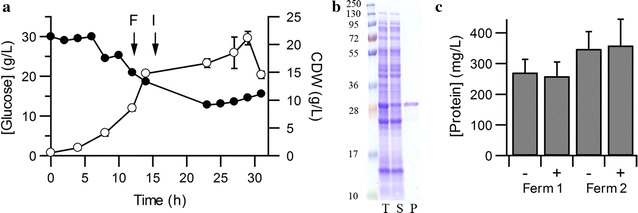
Production of scFv IgA_1_. **a** Representative growth profile of K-12 strain BW25113 co-expressing coPDI, coErv1p and scFv IgA_1_. *Error bars* represent the standard deviation from 3 samples. *F* indicates feeding started, *I* indicates induction. Cells were harvested and protein purified from the last time point shown. **b** Coomassie stained reducing SDS-PAGE analysis of produced proteins: molecular marker, total *E. coli* lysate (T), soluble protein fraction (S) and IMAC purified scFv. **c** Bradford analysis of sheared (+) and non-sheared (−) cells from two fermentations of scFv IgA_1_ (n = 6). There was no significant difference in protein concentration between sheared and non-sheared cells indicating the *E. coli* strains with CyDisCo components are robust

As proof of concept of the wider applicability of CyDisCo an alternative and very different methodology set for fermentation was used for scFv IgA_1_. scFv IgA_1_ producing fermentations were grown at 30 °C and a constant feeding rate was applied (Fig. 2a). After induction about half of the scFv was produced in a soluble form in the cytoplasm of *E. coli* and a purified yield of 139 ± 10 mg/L (average 6.6 mg/g CDW) was obtained (Fig. 2b). scFv IgA_1_ purified from fermentation showed the expected molecular weight (Table 1) for the protein with disulfides present, while an Ellman’s assay indicated 0.03 free thiol groups per protein. Preliminary results with other scFv (data not shown) indicate scFv-dependent yields up to 0.6 g/L, similar to the scFv-dependent yields seen in smaller scale cultures [16]. This possibly arises due to the difference in solubility of the scFv with scFv solubilities being highly variable due to the complementary determining regions (CDRs).

Upstream and downstream processing places stress on the cells and any system used on an industrial scale needs to be robust. To examine the robustness of cells containing the CyDisco system Bradford analysis of sheared vs non-sheared cells harvested after 32 h of fermentation of cultures expressing scFv IgA_1_ was used. The results showed no difference in total soluble protein released from the cells (Fig. 2c). These data indicate that the *E. coli* strains with CyDisCo are robust and not sensitive to shearing forces normally generated during upstream and downstream processing.

## Discussion

*Escherichia coli* is a first-choice host for recombinant protein production, however the production of proteins with disulfide bonds is limited in this host. The cytoplasm of *E. coli* is a reducing environment hence the formation of disulfide bonds is favored only in the periplasm, a compartment which can be problematic for the efficient production of recombinant proteins. When a heterologous protein is expressed in the cytoplasm of *E. coli* the unfavorable folding environment can lead to aggregation and formation of insoluble inclusion bodies. Although inclusion bodies can be solubilized and re-folded in vitro, the procedures often require extensive and expensive protein specific optimization. In addition, the final yield of natively folded active protein can be low and the separation of natively folded and misfolded or partially folded protein can be problematic since their biophysical properties e.g. isoelectric point are often very similar. In addition, methods that are developed in research facilities on the shake flask scale are often infeasible on larger scale.

While systems based on disruption of the cytoplasmic pathways for disulfide reduction have been reported and marketed for the production of disulfide bond containing proteins, these strains require the use of complex additives e.g. yeast extract and/or soytone.

Previously we showed that *E. coli* strains expressing CyDisCo components along with a protein of interest allow efficient disulfide bond formation and protein folding to native state for proteins including alkaline phosphatase, AppA [16], GH1 [19], BPTI, vtPA, resistin, Ero1α, CFS3, BMP4, catalytic light chain of enterokinase, IFNα2, IL-17 [18] and multiple scFv and Fab antibody fragments [17]. The yields of protein obtained reported could be up to 800-fold higher than in the absence of CyDisCo [18]. We also revealed that disruption of the reducing pathways in the cytoplasm is not necessary for soluble production of disulfide bonded proteins [16]. However, all published research is based on cultures cultivated on rich media, such as LB or EnPressoB, and all grown in deep well plates or in shake flasks. Such small scale cultivations may be sufficient for protein production for academic research, but are not apt for industrial scale. Growth conditions in shake flasks are difficult to measure and cannot be easily controlled and hence large batch-to-batch variations may occur. In contrast, production in bioreactors allows key variables such as pH, temperature, dissolved oxygen level and nutrient supply to be regulated. Chemically defined minimal media is preferred for large scale of protein production since it provides full control over media composition, avoiding a lot-to-lot variability of undefined components, it therefore assures reproducibility of the process and the quality of the final product. In addition, it is free of animal derived components. The slower growth profile of the cells on defined minimal media may be beneficial for protein folding, while it still allows implementation of fed-batch cultivations yielding high cell densities [21]. In the present research we scaled up cultivations.

Here we show that the limitation of *E. coli* cytoplasm for the production of recombinant disulfide-bonded proteins can be overcome by the introduction of CyDisCo components and that this system is feasible for scale-up to bioreactor cultures on chemically defined minimal media. In the present proof of concept research we scaled up cultivations of *E. coli* cultures producing recombinant proteins in presence of CyDisCo components to stirred tank bioreactors with expression under standard conditions 30 °C, pH = 7, pO_2_ = 30% on chemically defined, minimal media and the process was successful. Our K-12 (wild-type) strain cultures reached good cell densities and high protein yields: purified GH1 and IL-6 were around 1 g/L, while purified scFv IgA_1_ productivity reached 139 mg/L and avidin 71 mg/L, using two different standard starting fermentation protocols i.e. without protein specific optimization. In the case of the scFv IgA_1_ the glucose concentration was high through the fermentation and that may have repressed protein production. Protein and strain specific process optimization is likely to both increase the cell densities and protein productivity. To our knowledge this is the first report showing the efficient production of disulfide bond containing proteins in the cytoplasm of *E.coli* in chemically defined minimal media and our non-optimized yields of up to 1 g/L suggest it has potential industrial as well as academic use.

Our results contrast with the available data on soluble protein production in a bioreactor system in the cytoplasm of commercial available Δ*trxB/*Δ*gor* strains such as SHuffle or Origami. For example, Chung et al. [22] used SHuffle strain in fed-batch fermentation with rich media for the production of giant grouper growth hormone, however the protein was produced only in the form of inclusion bodies. Similarly Brüsehaber et al. [23] used the Origami strain with rich media for the production of pig liver esterase, but no soluble protein was obtained from fed-batch fermentation, only from batch fermentations. Lamppa et al. [24] were able to employ the SHuffle strain in fermentation for production of human lysozyme, however they managed to obtain soluble lysozyme only after co-expression of additional folding chaperones, Skp and Trigger factor resulting in the production of 21 mg/L of soluble lysozyme, far below the protein production levels reported here. In addition, the standard protocol for the use of SHuffle in fermentations [15] indicates that the strain requires complex additives (yeast extract and soytone), low concentrations of glycerol (below 2%) and low dissolved oxygen level (5–10%) in order to grow. Under the recommended cultivation conditions for fermentation SHuffle strains reach a final OD_600_ of only 25–30. SHuffle strains can be also cultivated on glucose as carbon source, but low dissolved oxygen level will cause organic acid accumulation and inhibition of growth [15].

The results of our study indicate that efficient production of valuable proteins such as GH1, IL-6, scFv IgA_1_ and avidin in a soluble folded form in the cytoplasm of *E. coli* is possible in chemically defined minimal media, although all of them have a long history of cumbersome attempts to produce them in active form. Growth hormone plays a key role in stimulating growth, reproduction and regeneration of cells. Historically it was extracted from cadaver pituitaries, but this led to several cases of Creutzfeld–Jacob disease, therefore its recombinant counterpart was of high interest. It is a non-glycosylated protein therefore there is a preference for production in prokaryotic cells. It was first synthetized in *E. coli* as inclusion bodies by Genentech and recombinant GH1 was accepted by the FDA in 1985 [25]. Today there are several GH1 pharmaceuticals produced in *E. coli* available on the market (Nutropin or Protropin (Genentech), Norditropin (Novo Nordisk), Genotropin (Pharmacia and Upjohn Company), Humatrope (Eli Lilly and Company) [26]), however, production is based on inclusion body formation, thus multiple methods for solubilization and re-folding have been developed. In contrast, there is no reported *E. coli* system we are aware of for production of IL-6 that would be economically feasible at larger scale. IL-6 is a human cytokine involved in a broad range of biological functions, e.g. acts as pro-inflammatory cytokine and anti-inflammatory myokine; it is associated with a number of inflammatory diseases, e.g. rheumatoid arthritis, atherosclerosis, diabetes. Its mature form has 2 sequential disulfide bonds and one N-glycosylation site. IL-6 expressed in *E. coli* cytoplasm as inclusion bodies requires intensive solubilization protocols [27]. IL-6 solubility in the cytoplasm has been significantly increased through fusion with tags e.g. NusA [28] and MBP [29]; however, a large proportion of the protein is lost during removal of the MBP-tag, resulting in a final yield of 270 mg/L [29]. Nausch et al. tested several approaches of IL-6 production, the most successful method produced 2.6 mg/L of active IL-6 in the cytoplasm of *E. coli* BL21 by co-expression with chaperones DnaK, DnaJ, GrpE, GroES, GroEL and lowering the cultivation temperature to 22 °C; authors report that this method was more successful than production in Origami 2 [30]. IL-6 targeted for secretion through hemolysin export apparatus via fusion with HlyA_S_ secretional signal yielded in 18 µg/L of fusion protein [31]. In contrast we report here 1.1 g/L of purified IL-6 from non-optimized fermentation.

ScFv constitute of an immunoglobulin variable light (VL) and variable heavy (VH) domain connected by a flexible linker; each domain is stabilized by one disulfide bond. ScFv are the smallest antibody fragments retaining the antigen binding capacity and specificity, therefore have potential applications in research, diagnostic and as therapeutic agents. In 2009 there were 19 scFv under clinical trials [32]. In comparison to the full length antibody scFv can penetrate tumor tissue more efficiently, while their clearance from the blood and retention time in non-target tissue is shorter. scFv lack the Fc region therefore they are less immunogenic than full length antibodies and do not require N-glycosylation, making them suitable for production in prokaryotic cells. Fv-fragments had already been produced in the periplasm of *E. coli* in 1988 [33], but prior to CyDisCo only scFv that do not require disulfide bonds for correct folding, and are thus soluble, were made in the cytoplasm of *E. coli* [34]. Previously using CyDisCo we produced a variety of scFv on shake flask scale in EnPresso-B media [17], where scFv IgA_1_ yielded 68 mg/L purified protein (7.9 mg/g CDW). Scaling up the production of this protein to a bioreactor on minimal media here increased the yield to 139 mg/L (6.6 mg/g CDW) through an increase in cell density.

Avidin is a glycoprotein synthesized in oviducts of avians, reptiles and amphibians. Avidin forms a non-covalent complex with biotin, with the strongest known affinity (Kd = 10^−15^ M) [35], making it useful for many research applications, including detection and purification of nucleic acids and proteins. However, avidin is highly glycosylated and has a high pI of 10–10.5 that contributes to nonspecific interactions and lectin binding. Deglycosylation of avidin decreases the pI to 6.3 and reduces lectin binding, thus increasing specificity for biotin without affecting affinity [36]; this form of avidin is called neutravidin. On an industrial scale avidin is purified from chicken egg white, in which it constitutes about 0.05% of total protein [37] (approximately 1.5–2 mg/egg). This production method is difficult to scale-up and results in batch-to-batch variation. Attempts to produce avidin in *E. coli* resulted in yields of 20 mg/L of active protein recovered from inclusion bodies [38] or 10 mg/L of soluble protein from periplasmic production [39]. Here we produced soluble aglycosylated avidin in the cytoplasm of *E. coli* on bioreactor scale with purified yields of 71 mg/L from a non-optimized expression system.

Currently expression of recombinant proteins in *E. coli* accounts for 30% of production of marketed pharmaceuticals [40], with the inability to carry out post-translational modifications causing it to lose its leading role to Chinese Hamster Ovary (CHO) cultures [41]. In contrast to bacterial cultures CHO have high nutritional requirements, grow slowly, are very fragile and instable; establishment and optimization of production strains for each protein of interest takes a long time correlating with high production costs [41]. Moreover, glycosylation patterns are heterogeneous [42] and the downstream processing requires the clearance of viruses [43]. Our results clearly show that the limitation related to formation of disulfide bonds in the cytoplasm of *E. coli* can be overcome by introduction of the CyDisCo system and that this can be applied in production of disulfide bonded proteins on larger scale in chemically defined minimal media. Our system can compete with CHO cultures for disulfide formation and has the potential to be a cheaper and more efficient method for pharmaceutical protein production.

## Conclusions

Here we show that *E. coli* K-12 strains with intact reducing pathways and co-expressing CyDisCo components are feasible to scale-up at bioreactor scale and can be applied for biosynthesis of valuable proteins containing disulfide bonds on a larger scale in chemically defined minimal media. Good cell densities and high yields of soluble protein were achieved using defined minimal media in glucose fed-batch cultures, without requiring process or strain optimization. Preliminary data suggests that CyDisCo can be employed in cultures utilizing glycerol as main carbon source as well; moreover, the CyDisCo system can be facilely transferred between *E. coli* strains.

## Methods

All chemicals used in this study, unless specified otherwise, were purchased from Sigma Aldrich and were of analytical grade.

### Strain and vectors

Two strains were used in this study: the parental strain of Keio collection single gene knock outs K-12 *BW25113* [rrnB*3* ΔlacZ*4787* hsdR514 Δ(araBAD)567 Δ(rhaBAD)568 rph-1] [44, 45] and K-12 *W3110* [F^−^ lambda^−^ IN(rrnD-rrnE)1 rph-1] [46, 47]. Both strains have the cytoplasmic reducing pathways intact.

Expression vectors (Table 2) were made by standard molecular biology techniques.

**Table 2:** Plasmids used in this study

Plasmid	Details	Selection	References
pMJS144	MH_6_M-mature human GH1, coErv1p, coPDI	Amp^R^	This study
pMJS145	MH_6_M-mature human IL-6, coErv1p, coPDI	Amp^R^	This study
pYU25	MH_6_M-mature hen avidin, coErv1p, coPDI	Amp^R^	This study
pJV84	scFv human IgA1-GSH_6_	Amp^R^	[16]
pMJS205	coErv1p, coPDI	Cm^R^	[16]

All plasmids except pMJS205 were pET23/Ptac based. pMJS205 is pLysS/Ptac based

The vector for expression of scFv IgA_1_ (pJV84) was a modified version of pET23 in which the T7 promoter was replaced with the *tac* promoter (Ptac), as described previously [17].

A polycistronic expression construct pMJS205 for codon optimized Erv1p and codon optimized mature PDI was made in modified pLysS vector as described previously [17].

pMJS144, pMJS145 and pYU25 are polycistronic vectors constructed for co-expression of three proteins: protein of interest with hexa-histidine tag on N-terminus, yeast sulfhydryl oxidase Erv1p and human protein disulfide isomerase (PDI). Genes encoding Erv1p (*Saccharomyces cerevisiae* Erv1p: Met1-Glu189) and PDI (human mature PDI: Asp18-Leu508) were synthesized codon optimized (co) for *E. coli* expression (GenScript). They were cloned *Nde*I/*Bam*HI into a modified pET23d vector [48]. The generated vectors (pMJS1 and pMJS2, respectively) were digested with *Xba*I/*Nde*I and the short fragments were replaced by annealed oligonucleotide pairs S1E1 for coErv1p and S1P1 for coPDI (S1E1f CTAGATTTATTATTTGATTCTATAAAGAAGGAGATAT; S1E1r TAATATCTCCTTCTTTATAGAATCAAATAATAAAT; S1P1f CTAGAATAATAAATCATAAGTAATAAGAAGGAGATAT; S1P1r TAATATCTCCTTCTTATTACTTATGATTTATTATT) to create vectors pMJS92 and pMJS96, respectively. The vector containing coErv1p was digested with *Bam*HI/*Xho*I and the small fragment was replaced with annealed oligonucleotide pair S2 (S2f GATCTATATGACTAGTATATTAATTGATCATA; S2r TCGATATGATCAATTAATATACTAGTCATATA). The resulting vector was digested with *Spe*I/*Bcl*I and the digested fragment was replaced with *Xba*I/*Bam*HI fragment from the coPDI containing vector pMJS96. The vector now containing coErv1p and coPDI was digested *Xba*I/*Bgl*I and the fragment was ligated into a *Spe*I/*Bgl*I digested modified pET23 vector containing Ptac instead of T7 promoter [17] and additional *Spe*I restriction site inserted after C-terminal His-tag using site directed mutagenesis, to generate vector pMJS143. The genes encoding for mature GH1 (Phe27–Phe217) and mature IL-6 (Val30-Met212) were amplified using IMAGE clones (http://www.imageconsortium.org) as templates and cloned *Nde*I/*Bam*HI into pMJS143 to generate pMJS144 and pMJS145. Gene encoding for mature hen avidin (Ala25-Glu152) was cloned *Nde*I/*Bam*HI from a codon optimized GenScript construct into pMJS143 to generate pYU25.

All plasmids were purified using the Gen-Elute HP Plasmid Miniprep Kit (Sigma Aldrich) and all DNA fragments were purified from agarose gels using the Gel/PCR DNA Fragments Extraction Kit (GeneAid), both according to the manufacturers’ instructions.

All generated constructs were sequenced to confirm that there are no errors in the cloned genes.

### Cultivation media

Liquid LB media contained per litre: 10 g NaCl, 10 g tryptone, 5 g yeast extract; additionally 15 g of agar was used for preparation of LB-agar plates.

Mineral salt medium (MSM) had the following composition (per liter): 2 g Na_2_SO_4_, 2.7 g (NH_4_)_2_SO_4_, 0.5 g NH_4_Cl, 14.0 g KHPO_4_, 3.6 g NaH_2_PO_4_ × H_2_O, 1.0 g (NH_4_)_2_ citrate [49].

After sterilization, the MSM was supplemented with sterile solutions of 100 µg/mL thiamine hydrochloride and 2 mL/L of trace element solution that included per liter: 0.5 g CaCl_2_ × 2H_2_O, 0.18 g ZnSO_4_ × 7H_2_O, 0.1 g MnSO_4_ × H_2_O, 20.1 g Na_2_-EDTA, 16.7 g FeCl_3_ × 6H_2_O, 0.16 g CuSO_4_ × 5H_2_O, 0.18 g CoCl_2_ × 6H_2_O [50]. MgSO_4_ was added to the final concentration of 2 mM at the start of the fermentation and again during the fermentation, as indicated below.

100 µg/mL of ampicillin and, where required, 35 µg/mL of chloramphenicol (i.e. for strains co-transformed with pMJS205) were used as selection markers in all LB-agar plates and liquid media, as well as in the feeding solutions.

### Fermentation cultures expressing GH1, IL-6 and avidin

*Escherichia coli* K-12 strains transformed with plasmid pMJS144, pMJS145 or pYU25 were stored in 20% glycerol at −70 °C; when needed the strains were streaked out from the glycerol stock onto LB-agar plates and incubated overnight at 37 °C. The next day a few colonies were used to seed 10 mL of LB media and incubated overnight (14 h) at 30 °C, 180 rpm. 1 mL of overnight culture was used to inoculate 200 mL of MSM (containing 40 g/L glucose), which was then incubated at 37 °C, 160 rpm for about 18 h. The MSM-based pre-cultures were applied to inoculate the batch of 0.6 L of sterile MSM containing 10 g/L of glucose or glycerol to a starting OD_600_ = 0.5.

For most fermentations the culture was incubated at 37 °C to accumulate the biomass before lowering to 30 °C prior to induction, but growth at a constant temperature of 30 °C was also tested. 2 mM MgSO_4_ was added during the fermentation at each increase of ten in the OD_600_ value. Fed-batch phase started automatically when the dissolved oxygen level rose to 40% corresponding to depletion of glucose/glycerol in the media. The feeding solution for fed-batch cultivations was based on fully formulated MSM with the required antibiotics and 300 g/L glucose/glycerol; the carbon source solution was delivered to the bioreactor with exponential feeding rate adjusted to specific growth rate µ = 0.15 per hour, which is much lower than the maximum growth rate on glucose. Growth at lower specific growth rate helps to avoid excretion of acetic acid that is one of major constraints of recombinant protein production during aerobic growth of *E. coli* [21, 51].

The exponential feeding profile was calculated according to the following equation:

$${\text{F}}({\text{t}}) = \frac{{{\upmu} V_{0} X_{0} {\text{e}}^{{{\upmu} t}} }}{{S_{0} (Y_{X/S} + m)}}$$

where *F(t)* is the feeding rate [g/h], *μ* is the specific growth rate [h^−1^], *V*_*0*_ is the culture volume [L] and *X*_*0*_ is the CDW [g/L] at the end of the batch phase, *t* is the time after feed start [h], *S*_*0*_ [g/g] is the substrate concentration in the feeding solution, *Y*_*x/s*_ is the yield coefficient [g of cell dry weight per g of glucose]: Y_x/s_ in all calculations was 0.4 g/g, *m* is the maintenance coefficient (0.025 g/g [21]).

The cultivation temperature was decreased to 30 °C 0.6–2 h prior to the induction with 0.1 mM IPTG. The cultivations were maintained for about 5 h after induction.

For protein analysis samples of the culture were collected from the bioreactor at the end of fermentation; samples were centrifuged, the supernatant was discarded and the pellets were frozen at −20 °C. For downstream processing the pellets were thawed, resuspended in the lysis buffer [50 mM sodium phosphate buffer, 0.1 mg/mL chicken-egg lysozyme and 40 µg/mL of DNase (Roche), pH = 7.4]. Lysis was performed by freeze-thawing.

Cultivations of strains expressing GH1, IL-6 and avidin were performed in the Greta multifermentor (Belach Bioteknik, Sweden) that allows to operate six 1 L stirred tank reactors in parallel. The pH value was adjusted to pH = 7.0 by titrating 25% ammonium hydroxide. Oxygen level was maintained at 30% of air saturation by cascade regulation of stirrer speed (800–1400 rpm) and air flow (0.4–2 L/min). Sigma's Aldrich AntiFoam 204 was used to avoid foaming.

Cell growth was followed by measurement of optical density and cell dry weight. The optical density was measured with spectrophotometer (Thermo Electron Corporation, Genesys 10 UV) at a wavelength of 600 nm. For determination of CDW three 1 mL aliquots of culture were centrifuged (16,000×*g* for 3 min at 4 °C) in pre-weighed Eppendorf tubes; the supernatant was transferred to other tube for glucose analysis and the pellets were dried at 60 °C for 48 h and the weight was measured again.

Glucose concentration in the media was analyzed with YSI 2700 Select Biochemical Analyzer (YSI Inc., Yellow Springs, USA).

### Fermentation cultures expressing scFv IgA_1_

*Escherichia coli* K-12 strain co-transformed with plasmids pJV84 and pMJS205 was stored as a 20% glycerol stock at −70 °C and was streaked out onto LB-agar plate and incubated at 37 °C overnight. The next day a few colonies were used to seed 10 mL of LB media and incubated overnight (16 h) at 30 °C, 250 rpm. 1 mL of LB pre-culture was used to inoculate 200 mL of MSM (containing 30 g/L of glucose), which was then incubated at 30 °C, 250 rpm, 24 h. The next day the MSM-based culture was used to inoculate batch of 0.9 L of MSM media containing 30 g/L of glucose to a starting OD 600 nm = 0.5.

scFv IgA_1_ expressing cultures were grown in Multifors system (Infors Ltd, Reigate, UK) that allows to handle 4 × 1 L stirred tank reactors in parallel (900 mL working volume). Appropriate antibiotics were added to the defined medium prior to inoculation. 2 mM MgSO_4_ was added during the fermentation at inoculation, 8 h after induction and 10 h after induction. Fermenters were inoculated with approx. 50 mL cultures to an OD_600_ = 0.4. During fermentation the dissolved oxygen level was maintained at 30% of air saturation by using air or air/oxygen mixture as required and by automatic regulation of the stirrer rate (500–1100 rpm). The cultivation temperature was maintained at 30 °C. The pH was held at 7.0, adjusted with 5% v/v solution of ammonium hydroxide. Polypropylene glycol P 2000 was applied to prevent foaming.

The process was in the batch mode for 12 h and the fed-batch started when the biomass concentration reached OD_600_ around 12 (equivalent to a CDW of about 8 g/L). The feeding solution was based on fully formulated MSM and contained 650 g/L of glucose. A constant feeding rate of 4 mL/h was applied.

The cultures were induced with 1 mM IPTG at an OD _600_ approximately 20 (14 h of cultivation). The cells were harvested 18 h post induction.

Fermentation samples were collected from the bioreactor, centrifuged at 3220×*g*, the supernatant was discarded, and the pellets were resuspended in the lysis buffer [50 mM sodium phosphate buffer, 0.1 mg/mL chicken-egg lysozyme and 20 µg/mL of DNase (Roche), pH = 7.4], to equivalent of culture OD_600_ = 10. Lysis was performed by freeze-thawing.

Cell density was monitored by measurements of OD at 600 nm and samples were taken to determine CDW. For determining cell dry weight 1 mL of culture in replicates was collected to pre-dried (100 °C, 24 h) and pre-weighed 1.5 mL Eppendorf tubes; the samples were centrifuged 10 min at 16,000×*g*. Cell pellets were dried at 100 °C for 24 h and tubes re-weighed.

Concentration of glucose in the fermentation broth was monitored on BioProfile FLEX Analyzer (Nova Biomedical): 1 mL samples of the culture were collected from the bioreactor, centrifuged 5 min at 16,000×*g* and the supernatants were used for the analysis.

### Robustness measurements

Culture samples were collected from bioreactors after 32 h of cultivation; a rotating disk shear device [52] was used to determine the relative robustness of *E. coli* cells [53]. 20 mL of cell broth were exposed for 20 s to a rotation speed of 233 revolutions per second in the device, which corresponds to an energy dissipation rate of 0.75 × 10^6^ W/kg [52]. This represents the energy normally generated in high shear producing devices such as pumps and process scale centrifuges. Pre and post-shearing samples were centrifuged at 17,000×*g* for 10 min, the supernatants collected and frozen at −20 °C. The supernatants were thawed and the total protein content in sheared and non-sheared samples was measured via Bradford assay (Thermo Scientific, IL, USA) in 96 well plate at A 595 nm (Tecan Safire2, Tecan, Reading, UK).

### Protein purification

Hexa-histidine tagged proteins were purified via immobilized metal affinity chromatography (IMAC) using either prepacked HiTrap IMAC columns (GE Healthcare; GH1 or IL-6) or HisPur Cobalt Superflow Agarose Resin (Thermo Scientific; GH1, scFv IgA_1_ or avidin).

For HiTrap IMAC, a 5 mL column was washed with 15 mL of water, loaded with 10 mL NiCl_2_, washed with 15 mL water and equilibrated with 2 × 15 mL of 20 mM phosphate buffer (pH 7.3). The lysates of the cells were thawed at room temperature, centrifuged at 3220×*g*, 20 min, 4 °C and the supernatants were loaded onto the resin. The resin was then washed with 10 mL of 20 mM phosphate buffer (pH 7.3) followed by 20 mL of wash buffer (20 mM sodium phosphate, 50 mM imidazole, 0.3 M sodium chloride; pH 7.3), then 15 mL of 20 mM phosphate buffer (pH 7.3). Elution was either with elution buffer 1 (20 mM phosphate buffer, 50 mM EDTA; pH 7.3) or in parallel samples elution buffer 2 (20 mM phosphate buffer, 250 mM imidazole). Circa 1 mL elution fractions were collected, analysed by absorbance measurements at 280 nm and fractions containing protein pooled. No differences were observed in either the yields obtained or the purity of the eluted material with the two elution methods.

For HisPur Cobalt Superflow Agarose Resin, 1 mL of resin was loaded onto the gravity-flow column, washed with 2 × 5 mL of water and equilibrated with 2 × 5 mL of 50 mM phosphate buffer (pH 7.4). The lysates of the cells were thawed at room temperature, centrifuged at 3220×*g*, 20 min, 4 °C and the supernatants were loaded onto the resin and incubated 5 min. The resin was equilibrated with 5 mL of 50 mM phosphate buffer (pH 7.4) and washed with 4 × 5 mL of wash buffer (50 mM sodium phosphate, 15 mM imidazole, 0.3 M sodium chloride; pH 7.4), equilibrated again with 5 mL of 50 mM sodium phosphate (pH 7.4) before elution with 4 × 1 mL of elution buffer (50 mM phosphate buffer, 50 mM EDTA buffer; pH 7.4).

After IMAC the purified protein samples were desalted on PD-10 columns (GE Healthcare) according to the manufacturer’s instructions into either 20 mM phosphate buffer pH 7.3 or 50 mM phosphate buffer pH 7.4. The protein concentration was determined via absorbance measurements at 280 nm. Total cell lysate, soluble protein fraction and purified protein samples were analyzed on reducing SDS-PAGE stained with Coomassie brilliant blue.

### Protein analysis

Ellman’s assay for free thiol content was performed under denaturing conditions: the purified protein sample was mixed with 50 mM Tris buffer (pH 8.0), 6 M guanidine hydrochloride to a final concentration of 2 M and 0.073 mg/mL of Ellman’s reagent; the samples were incubated at room temperature for 15 min and the absorbance was monitored at 412 nm compared to a no protein control sample. The free thiol content was calculated using a molar extinction coefficient of 13,600 M^−1^ cm^−1^.

Reverse phase high pressure liquid chromatography (rpHPLC) analysis was performed on an ÄKTA FPLC system (GE Healthcare) using a μRPC C2/C18 ST 4.6/100 column: The column was pre-equilibrated in buffer A (0.065% trifluoroacetic acid), before a 50 μL sample was loaded to the system using automatic sample injection. The elution gradient used was 0-100% buffer B (90% acetonitrile, 0.05% trifluoroacetic acid) over 8 column volumes. 0.5 mL fractions were collected with a flow rate of 0.3 mL/min. All buffers were filtered and degassed before use.

Molecular masses were measured by LCMS with an Aquity UPLC-system (Waters) connected to a Synapt G1 Q-ToF—type mass spectrometer. The analytical column was a BEH 300 C4, 2.1 × 100 mm (Waters) run at 0.4 mL/min using a gradient from 3% acetonitrile in water/0.1% formic acid to 43% acetonitrile over 1 min. Samples were acidified with trifluoroacetic acid to about 0.5% v/v and 5 µL was injected. The mass spectrometer was operated in sensitivity mode with lock mass corrected 1 s scans in continuous mode for m/z 100–2000. Capillary voltage was 3.5 kV, cone voltage 30 V. Baseline was subtracted and mass spectra were deconvoluted with MaxEnt1.

## Additional file

**Additional file 1.** Protein sequences. **Figure S1.** Production of GH1 in alternative strain and on alternative carbon source. **Figure S2.** Production of IL-6. Panel A) Growth profile of BW25113 strain expressing IL-6 in glucose fed-batch culture. Error bars represent the standard deviation from 3 samples. F indicates feeding started, I indicates induction. Cells were harvested and protein purified from the last time point shown. Panel B). Coomassie stained reducing SDS-PAGE analysis of produced proteins: molecular marker, total *E. coli* lysate (T), soluble protein fraction (S) and IMAC purified IL-6. Panel E) rpHPLC analysis of purified IL-6: comparison of IL-6 produced in fed-batch fermentation (red) and in shake flask (black). **Figure S3.** Production of avidin. Panel A) Growth profile of BW25113 strain expressing avidin in glucose fed-batch culture. Error bars represent the standard deviation from 3 samples. F indicates feeding started, I indicates induction. Cells were harvested and protein purified from the last time point shown. Panel B). Coomassie stained reducing SDS-PAGE analysis of produced proteins: molecular marker, total *E. coli* lysate (T), soluble protein fraction (S) and IMAC purified avidin.

## Abbreviations

CDW: cell dry weight
CDRs: complementary determining regions
CyDisCo: cytoplasmic disulfide bonds formation in the cytoplasm of *E. coli*
co: codon optimized
CHO: Chinese hamster ovary
GH1: growth hormone 1
IL-6: interleukin 6
IMAC: immobilized metal affinity chromatography
MSM: mineral salt media
PDI: protein disulfide isomerase
POI: protein of interest
rpHPLC: reverse phase high pressure liquid chromatography
scFv: single chain variable domain of an antibody
wt: wild type
