# Supplementary material for: Efficient soluble expression of disulfide bonded proteins in the cytoplasm of Escherichia coli in fed-batch fermentations on chemically defined minimal media
Source: Microb Cell Fact. 2017 Jun 15;16:108. doi: 10.1186/s12934-017-0721-x (PMC5471842; doi:10.1186/s12934-017-0721-x)

**Protein sequences**

**Growth hormone 1** (N-terminal his-tagged mature human somatotropin; UniProtKB - P01241)

MHHHHHHMFPTIPLSRLFDNAMLRAHRLHQLAFDTYQEFEEAYIPKEQKYSFLQNPQTSLCFSESIPTPSNREETQQKSNLELLRISLLLIQSWLEPVQFLRSVFANSLVYGASDSNVYDLLKDLEEGIQTLMGRLEDGSPRTGQIFKQTYSKFDTNSHNDDALLKNYGLLYCFRKDMDKVETFLRIVQCRSVEGSCGF

**Interleukin 6** (N-terminal his-tagged mature human protein; UniProtKB - P05231)

MHHHHHHMVPPGEDSKDVAAPHRQPLTSSERIDKQIRYILDGISALRKETCNKSNMCESSKEALAENNLNLPKMAEKDGCFQSGFNEETCLVKIITGLLEFEVYLEYLQNRFESSEEQARAVQMSTKVLIQFLQKKAKNLDAITTPDPTTNASLLTKLQAQNQWLQDMTTHLILRSFKEFLQSSLRALRQM

**scFv IgA_1_** (C-terminal his-tagged human IgA_1_ anti-beta tubulin; PDB entry 3M8O) MDIVMTQSPLSLSVTPGEPASISCRSSQSLLRRDGHNDLEWYLQKPGQSPQPLIYLGSTRASGVPDRFSGSGSGTDFTLKIIRVEAEDAGTYYCMQNKQTPLTFGQGTRLEIKGASGGGGSGGGGSGGGGSSEVQLVESGGGLVQPGGSLKLSCAASGFTLSGSNVHWVRQASGKGLEWVGRIKRNAESDATAYAASMRGRLTISRDDSKNTAFLQMNSLKSDDTAMYYCVIRGDVYNRQWGQGTLVTVSSGSHHHHHH

**Avidin** (N-terminal his-tagged mature chicken protein; UniProtKB - P02701)

MHHHHHHMARKCSLTGKWTNDLGSNMTIGAVNSRGEFTGTYITAVTATSNEIKESPLHGTQNTINKRTQPTFGFTVNWKFSESTTVFTGQCFIDRNGKEVLKTMWLLRSSVNDIGDDWKATRVGINIFTRLRTQKE

**Figure S1. Production of GH1 in alternative strain and on alternative carbon source.** Panel A) Growth profile of W3110 strain in glucose-based fed-batch culture. Panel B) Growth profile of BW25113 strain in glycerol-based fed-batch culture. In both panels A and B the error bars represent the standard deviation from 3 samples. F indicates feeding started, I indicates induction. Cells were harvested and protein purified from the last time point shown. Panels C-E) Coomassie stained reducing SDS-PAGE analysis of produced proteins: molecular marker, total *E. coli* lysate (T), soluble protein fraction (S) and IMAC purified GH1 from protein produced in BW25113/glucose (panel C), W3110/glucose (panel D) and BW25113/glycerol (panel E).


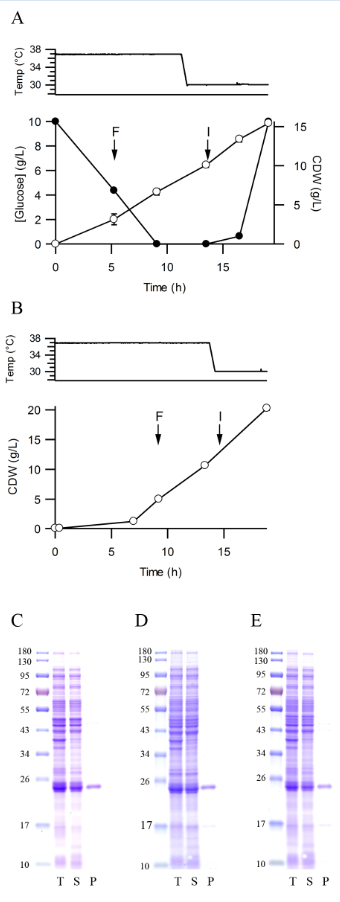


**Figure S2. Production of IL-6.** Panel A) Growth profile of BW25113 strain expressing IL-6 in glucose fed-batch culture. Error bars represent the standard deviation from 3 samples. F indicates feeding started, I indicates induction. Cells were harvested and protein purified from the last time point shown. Panel B). Coomassie stained reducing SDS-PAGE analysis of produced proteins: molecular marker, total *E. coli* lysate (T), soluble protein fraction (S) and IMAC purified IL-6. Panel E) rpHPLC analysis of purified IL-6: comparison of IL-6 produced in fed-batch fermentation (red) and in shake flask (black).


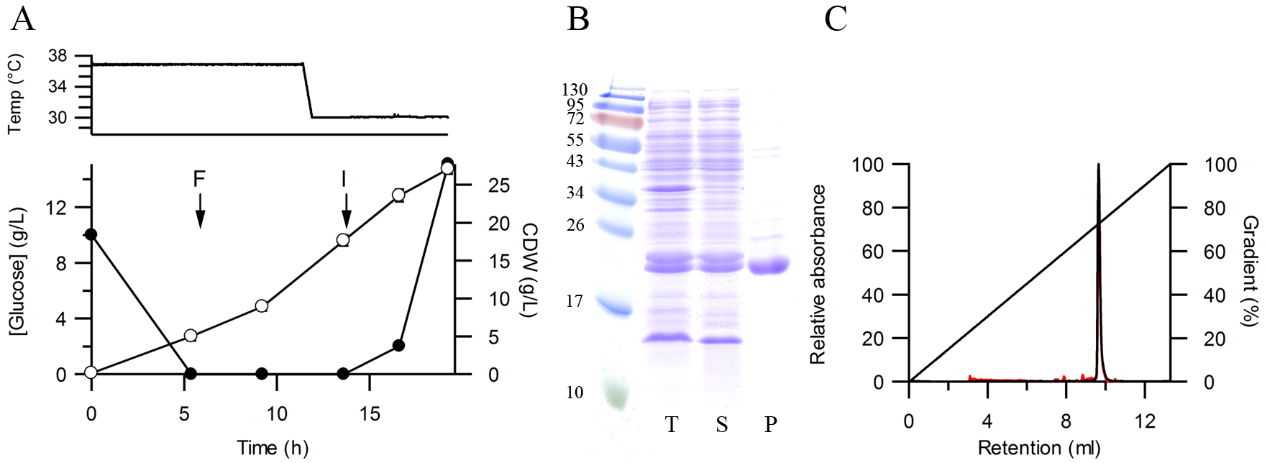


**Figure S3. Production of avidin.** Panel A) Growth profile of BW25113 strain expressing avidin in glucose fed-batch culture. Error bars represent the standard deviation from 3 samples. F indicates feeding started, I indicates induction. Cells were harvested and protein purified from the last time point shown. Panel B). Coomassie stained reducing SDS-PAGE analysis of produced proteins: molecular marker, total *E. coli* lysate (T), soluble protein fraction (S) and IMAC purified avidin.


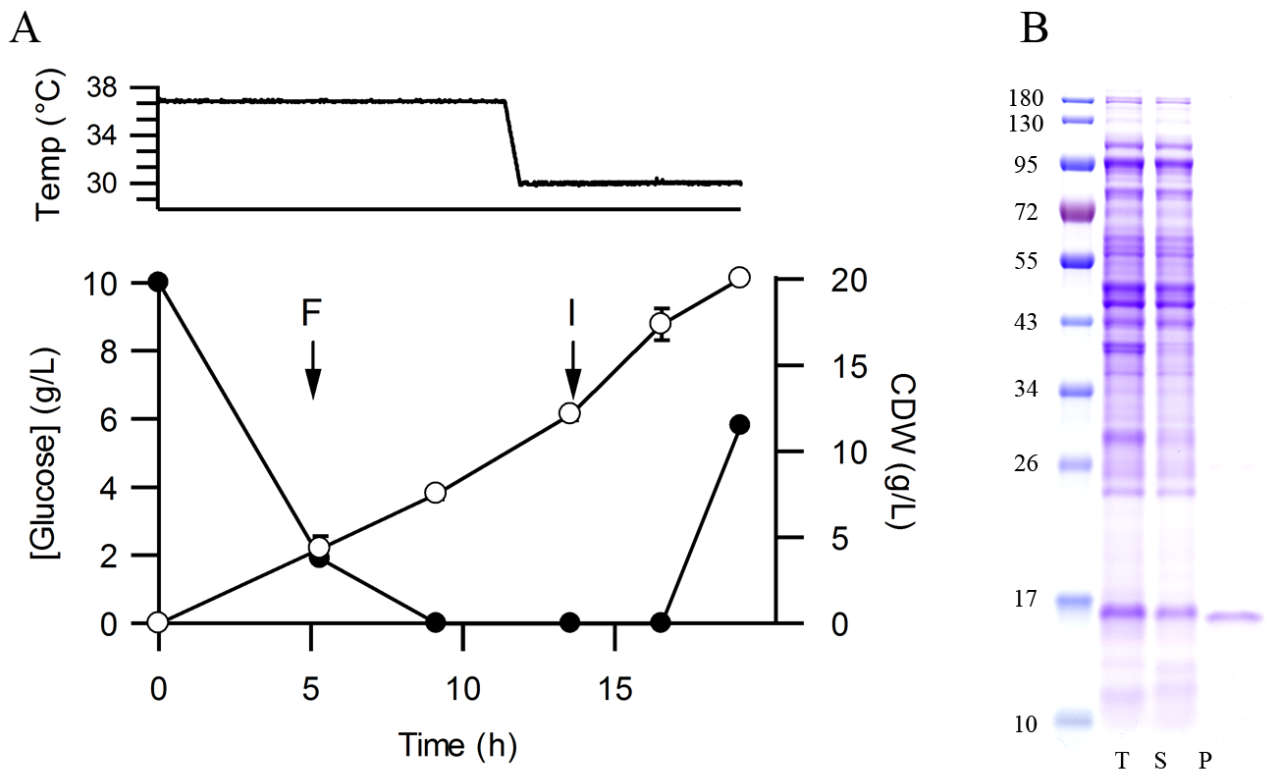

Supplement: Supplementary file 1 — Additional file 1. Protein sequences. Figure S1. Production of GH1 in alternative strain and on alternative carbon source. Figure S2. Production of IL-6. Panel A) Growth profile of BW25113 strain expressing IL-6 in glucose fed-batch culture. Error bars represent the standard deviation from 3 samples. F indicates feeding started, I indicates induction. Cells were harvested and protein purified from the last time point shown. Panel B). Coomassie stained reducing SDS-PAGE analysis of produced proteins: molecular marker, total E. coli lysate (T), soluble protein fraction (S) and IMAC purified IL-6. Panel E) rpHPLC analysis of purified IL-6: comparison of IL-6 produced in fed-batch fermentation (red) and in shake flask (black). Figure S3. Production of avidin. Panel A) Growth profile of BW25113 strain expressing avidin in glucose fed-batch culture. Error bars represent the standard deviation from 3 samples. F indicates feeding started, I indicates induction. Cells were harvested and protein purified from the last time point shown. Panel B). Coomassie stained reducing SDS-PAGE analysis of produced proteins: molecular marker, total E. coli lysate (T), soluble protein fraction (S) and IMAC purified avidin. [file 12934_2017_721_MOESM1_ESM.docx]
